# Identifying biologically implausible values in big longitudinal data: an example applied to child growth data from the Brazilian food and nutrition surveillance system

**DOI:** 10.1186/s12874-024-02161-1

**Published:** 2024-02-15

**Authors:** Juliana Freitas de Mello e Silva, Natanael de Jesus Silva, Thaís Rangel Bousquet Carrilho, Elizabete de Jesus Pinto, Aline Santos Rocha, Jéssica Pedroso, Sara Araújo Silva, Ana Maria Spaniol, Rafaella da Costa Santin de Andrade, Gisele Ane Bortolini, Enny Paixão, Gilberto Kac, Rita de Cássia Ribeiro-Silva, Maurício L. Barreto

**Affiliations:** 1 Centre for Data and Knowledge Integration for Health, Gonçalo Moniz Institute, Oswaldo Cruz Foundation, Salvador, BA Brazil; 2 ISGlobal, Hospital Clínic. Universitat de Barcelona, Barcelona, Spain; 3 Nutritional Epidemiology Observatory, Josué de Castro Nutrition Institute, Federal University of Rio de Janeiro, Rio de Janeiro, RJ Brazil; 4 Department of Obstetrics and Gynaecology, Faculty of Medicine, University of British Columbia, Vancouver, BC Canada; 5 Federal University of Recôncavo da Bahia, Santo Antônio de Jesus, BA Brazil; 6 Food and Nutrition Coordinating Unit, Ministry of Health, Brasília, DF Brazil; 7 London School of Hygiene & Tropical Medicine, London, UK; 8 School of Nutrition, Federal University of Bahia, Av. Araújo Pinho, nº 32, Canela, Salvador, Bahia, CEP: 40.110–150 BA Brazil; 9 Institute of Collective Health, Federal University of Bahia, Salvador, BA Brazil

**Keywords:** Biologically implausible values, Longitudinal growth data, Outliers, Outlier identification, Anthropometry, Child’s growth, Big data

## Abstract

**Background:**

Several strategies for identifying biologically implausible values in longitudinal anthropometric data have recently been proposed, but the suitability of these strategies for large population datasets needs to be better understood. This study evaluated the impact of removing population outliers and the additional value of identifying and removing longitudinal outliers on the trajectories of length/height and weight and on the prevalence of child growth indicators in a large longitudinal dataset of child growth data.

**Methods:**

Length/height and weight measurements of children aged 0 to 59 months from the Brazilian Food and Nutrition Surveillance System were analyzed. Population outliers were identified using z-scores from the World Health Organization (WHO) growth charts. After identifying and removing population outliers, residuals from linear mixed-effects models were used to flag longitudinal outliers. The following cutoffs for residuals were tested to flag those: -3/+3, -4/+4, -5/+5, -6/+6. The selected child growth indicators included length/height-for-age z-scores and weight-for-age z-scores, classified according to the WHO charts.

**Results:**

The dataset included 50,154,738 records from 10,775,496 children. Boys and girls had 5.74% and 5.31% of length/height and 5.19% and 4.74% of weight values flagged as population outliers, respectively. After removing those, the percentage of longitudinal outliers varied from 0.02% (<-6/>+6) to 1.47% (<-3/>+3) for length/height and from 0.07 to 1.44% for weight in boys. In girls, the percentage of longitudinal outliers varied from 0.01 to 1.50% for length/height and from 0.08 to 1.45% for weight. The initial removal of population outliers played the most substantial role in the growth trajectories as it was the first step in the cleaning process, while the additional removal of longitudinal outliers had lower influence on those, regardless of the cutoff adopted. The prevalence of the selected indicators were also affected by both population and longitudinal (to a lesser extent) outliers.

**Conclusions:**

Although both population and longitudinal outliers can detect biologically implausible values in child growth data, removing population outliers seemed more relevant in this large administrative dataset, especially in calculating summary statistics. However, both types of outliers need to be identified and removed for the proper evaluation of trajectories.

**Supplementary Information:**

The online version contains supplementary material available at 10.1186/s12874-024-02161-1.

## Background

Nutrition and food surveillance systems are essential resources for monitoring child growth indicators. These indicators can help planning public policies and actions toward children’s health [1, 2]. The most common child growth indicators (e.g., stunting, wasting, underweight, and overweight) are based on length/height and weight measurements according to age. They are commonly collected in healthcare services and used in public healthcare systems [3].

However, it is important to evaluate the quality of the anthropometric measurements available in these systems. Errors during data collection, documentation, and data entry into electronic systems can happen. In addition, measuring length in children under two years of age can be another challenge for many healthcare professionals [4, 5]. Identifying records with biologically implausible values (BIVs) is particularly important when working with routine electronic record data, as those may impact child growth indicators [6].

The methodological process of identifying BIVs has been investigated recently, but studies with large longitudinal datasets are scarce [7–9]. In cross-sectional studies, methods to detect BIVs are usually based on cutoffs defined according to a reference population. For example, the World Health Organization (WHO) recommends identifying biologically implausible child growth values based on the child growth standards [13].

In longitudinal studies, each child may have multiple anthropometric measurements collected at different time points or ages. Those sequences of measurements can be used as another source of information to evaluate the plausibility of the anthropometric measurements. Thus, it is possible to identify BIVs both cross-sectionally and longitudinally [11]. In general, BIVs flagged based on the sample’s distribution or according to external references are considered population outliers (POs), and measurements flagged based on an individual’s trajectory can be referred to as longitudinal outliers (LOs) [8].

Some studies have argued that identifying and removing POs alone may not be enough to clean longitudinal anthropometric data, and that identifying and considering LOs can be needed as well [7–9, 11, 12]. However, this statement is often based on data from studies with small sample sizes. Thus, our study aims to evaluate the impact of identifying and removing POs and the additional value of identifying and removing LOs in a large longitudinal dataset of child anthropometric measurements from the Brazilian Food and Nutrition Surveillance System (SISVAN). We also aim to compare the role of removing flagged BIVs on the child growth trajectories and on the prevalence of child growth indicators.

## Methods

### Data source

We analyzed individual-level data of children under five years old who were followed up in the Unified Health System’s (SUS) primary healthcare services between 2008 and 2017 and registered in the SISVAN. Monitoring nutritional status is part of the SISVAN, which consists of continuously evaluating the food and nutritional status of the Brazilian population. Healthcare professionals routinely carry out the data collection, entry, and initial analysis of anthropometric data of subjects using public services throughout the life course [13].

The SISVAN data were obtained from the Ministry of Health and accessed, processed, and analyzed in the Centre for Data and Knowledge Integration for Health (CIDACS, Oswaldo Cruz Foundation) [14]. For this study, authorized researchers accessed and analyzed only de-identified data.

### Study variables

Length/height (cm) and weight (kg) data were collected according to technical standards established by the Brazilian Ministry of Health for data collection in public health services [15]. Then, these data were retrieved from the SISVAN records for children under 59 months old. Length/height-for-age (HAZ) and weight-for-age (WAZ) z-scores were calculated based on the WHO child growth standards [16]. Age (in months) was calculated considering the difference between the date of visit to the primary care unit and the birth date. Sex was registered in the first visit.

The child growth indicators comprised length/height-for-age z-scores (L/HAZ) and weight-for-age z-scores (WAZ). L/HAZ was classified as severe stunting (L/HAZ < -3), moderate stunting (L/HAZ ≥ -3 and < -2), and adequate (L/HAZ > -2). WAZ classifications were severe underweight (WAZ < -3), moderate underweight (WAZ ≥ -3 and < -2), adequate (WAZ ≥ -2 and ≤ + 2), and overweight (WAZ > + 2) [16].

### Data cleaning and statistical analysis

The data were processed and analyzed using Stata version 15.1 and R version 3.6.0. We first removed duplicate records (defined in this study as those with the same identification number, birth date, date of visit, length/height, and weight). Duplicate records are common in the SISVAN because the data can be registered in three separate systems (the e-SUS, the Bolsa Familia system, and the SISVAN itself) and then consolidated into a unique dataset. After removing duplicates, we split the dataset into four sub-datasets to analyze length/height and weight by sex separately. We removed all observations with missing and/or negative values in the variables of interest (age, sex, length/height, and/or weight), and individuals with only one measurement of length/height or weight. This decision was made because those individuals were not eligible for the approach to flag LOs.

To identify POs, we adopted the WHO cutoffs for BIVs and flagged values based on the four child growth indicators: body-mass-index-for-age (BMIZ) < -5 and > + 5, L/HAZ < -6 and > + 6, WAZ < -6 and > + 5, and weight-for-length/height-for-age (WHZ) < -5 and > + 5 [10]. Since analysis for length/height and weight were performed separately, L/HAZ was used only for length/height data and, analogously, WAZ only for the weight dataset. The flagged values were then removed for the next step. Before flagging LOs, we performed another cleaning in the dataset to remove individuals who remained with only one measurement after removing POs. This additional cleaning was necessary because, although these individuals could contribute to the general mean with only one measurement, they increase the sample size contributing to an even smaller variance. However, they do not bring essential information. We were interested in the additional impact of the identification and removal of LOs in longitudinal data.

To flag LOs observations, the approach proposed by Boone-Heinonen et al. [8] was adopted with modifications. Mixed-effects models with random intercept were fitted considering restricted cubic splines for the time (age) variable. In the current study, models were fitted separately for length/height and weight, and also by sex, to accommodate sex-specific growth patterns. The model equation was given by

$$\begin{gathered}{Y_{ij}} = {\beta _0} + {b_{0i}} + \sum _{k = 1}^K{\gamma _k}{(ag{e_j} - {\varepsilon _k})^3} + {e_{ij}},{\text{for}}\,i \\ = 1,2, \ldots n\,and\,j = 1,2, \ldots ,{J_i},\,{\text{where}} \\ \end{gathered}$$

- ${Y}_{ij}$ is the value of the response variable for the i-th subject and the j-th measurement, n is the number of subjects, ${J}_{i}$ is the number of measurements for the i-th subject and $N = {\sum }_{i=1}^{n}{J}_{i}$ is the total number of measurements;
- ${\beta }_{0}$ is the intercept, representing an overall length or weight at birth;
- ${b}_{0i}$ is the i-th subject-specific random effect, representing the length or weight at birth;
- $\gamma = ({\gamma _1},{\gamma _2}, \ldots ,{\gamma _K})$ is the vector of coefficients associated with the cubic splines;
- $\varepsilon = ({\varepsilon _1},{\varepsilon _2}, \ldots ,{\varepsilon _K})$ is the vector of knots;
- $K$ is the number of knots;
- ${e}_{ij}$ is the normally distributed random error with mean zero and unknown variance ${\sigma }^{2}$.

The main changes between the approach used by Boone-Heinonen et al. [8] and the present one concern the decision regarding (i) the number and position of the knots used in the restricted cubic splines to model the variation of length/height and weight according to age and the type of residual used to flag LOs, and (ii) the residual used to flag LOs. Five knots (K = 5) located at 2, 6, 12, 24, and 58 months were used to model length/height. Four knots placed at 3, 6, 12, and 58 months were used to model weight. These knots were selected based on the WHO child growth curves inflection points [16].

After fitting the model, scaled residuals were obtained via the following equation:

$${R}_{ij}= \frac{{e}_{ij}}{\sqrt{\widehat{Var\left({Y}_{ij}\right)}}}$$

where ${e}_{ij} = {y}_{ij} - {\widehat{y}}_{ij}$, that is, the difference between the observed value of length/height or weight for the i-th subject at the j-th time point (age), and their respective fitted value. These residuals are simple, easily obtained, and able to account for the fitted values’ variance. After calculating the scaled residuals, we flagged LOs considering the following cutoffs: -3/+3, -4/+4, -5/+5, and − 6/+6.

Scaled residuals were selected due to the computational difficulty of extracting studentized residuals in a large dataset, as performed by Boone-Heinonen et al. [8]. The functions available in R and Stata for the extraction of studentized residuals did not work in the SISVAN large sample. A sensitivity analysis was performed with a random sample of 1,000 boys to compare the scaled, internally studentized, and externally studentized residuals from the same model. The results indicated that the number of observations flagged by each type of residual was similar (Table S1), which allowed us to keep the decision to work with scaled residuals.

The impact of removing BIVs flagged from several approaches, including POs and LOs, on the trajectories and prevalence of child growth indicators (HAZ and WAZ) was evaluated. We estimated the trajectories of length/height and weight using the mixed-effects models previously fitted. The prevalence of child growth indicators was calculated in the initial dataset, after removing POs, after removing children with only one measurement, and after removing both POs and LOs according to the different cutoffs.

In the initial exploration of the dataset, it was observed that a child’s series of length/height measurements presented decreasing value(s) in relation to the previous one(s) – a characteristic called “decreasing heights”. Decreasing linear growth is not biologically plausible in children aged until 59 months; however, small negative differences in heights between visits can arise due to measurements taken by different workers and/or by using different measurement devices. Thus, to consider these special cases, the difference between adjacent length/height measurements was calculated, and the observations were considered inaccurate if such a difference was lower than − 2 cm. A secondary analysis was conducted after removing POs by identifying and removing these “decreasing height” values. In this case, our strategy assumed that the first measurement was correctly performed/registered.

## Results

**Fig. 1:**
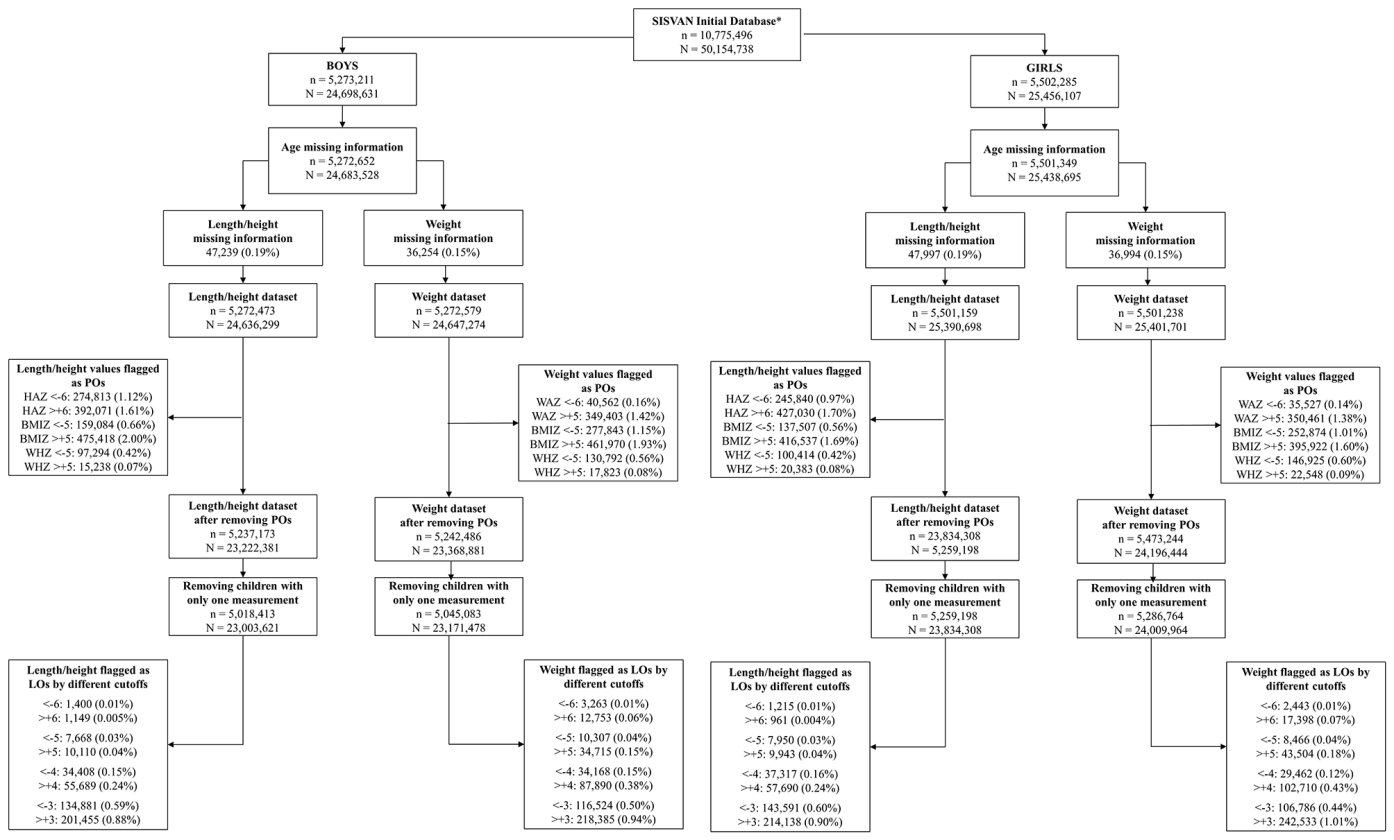
Flowchart for the construction of the datasets used for the assessment of the population and longitudinal outliers. Notes: ‘SISVAN initial database’ refers to the extracted database after removing duplicates and children with a single measurement. SISVAN: Brazilian Food and Nutrition Surveillance System, L/HAZ: length/height-for-age z-score, WAZ: weight-for-age z-score, n: number of children, N: number of measurements

The initial SISVAN dataset comprised 15,885,550 children and 55,264,792 measurements. After removing duplicates, missing data, and individuals with only one length/height or weight measurement, 24,636,299 length/height and 24,647,274 weight measurements for boys and 25,390,698 length/height and 25,401,701 weight measurements for girls were available for the analyses. Following the removal of POs and children with only one measurement, the dataset used for assessing LOs comprised 5,018,413 boys and 5,259,198 girls for length/height and 5,045,083 boys and 5,286,764 girls for weight (Fig. 1).

Boys and girls had 5.74% and 5.31% of the length/height and 5.19% and 4.74% of the weight measurements flagged as POs, respectively. Considering BMIZ, HAZ, and WAZ, there were more POs on the positive side (extreme) of the curve (WAZ > + 5 and L/HAZ > + 6) than in the negative part of the interval (WAZ and L/HAZ < -6). On the other hand, WHZ presented more POs on the negative side. Descriptive statistics show considerable changes in length/height, L/HAZ, weight, and WAZ for both sexes after the removing POs, especially in the means, minimum and maximum values (Table 1). Mean L/HAZ decreased from 15.40 to -0.36 for boys and from 11.50 to -0.29 for girls.

**Table 1:** Descriptive statistics for anthropometric variables before and after removing population outliers. Brazilian Food and Nutrition Surveillance System (SISVAN), 2008–2017. Notes: POs: population outliers, SD: standard deviation, L/HAZ: length/height-for-age z-score, WAZ: weight-for-age z-score

	Initial dataset		Dataset after removing POs		Dataset after removing children with only one measurement	
	Mean (SD)	Min; Max	Mean (SD)	Min; Max	Mean (SD)	Min; Max
**Boys**						
Age (months)	31.41 (16.55)	0.00; 59.00	31.47 (16.54)	0.00; 59.00	31.45 (16.53)	0.00; 59.00
Length/height (cm)	136.40 (5914.07)	0.00; 993,900.00	90.04 (14.54)	45.00; 120.00	90.02 (14.53)	45.00; 120.00
L/HAZ	15.40 (2034.79)	-32.10; 433,915.30	-0.36 (1.54)	-6.00; 6.00	-0.36 (1.53)	-6.00; 6.00
Number of measurements	4.68 (4.08)	2;145	4.43 (3.99)	1; 113	4.58 (4.01)	2; 113
Age (months)	31.41 (16.55)	0.00; 59.00	31.41 (16.54)	0.00; 59.00	31.38 (16.54)	0.00; 59.00
Weight (kg)	15.50 (141.90)	0.00; 147,000.00	13.60 (4.03)	1.70; 34.80	13.59 (4.02)	1.70; 34.70
WAZ	1.03 (65.04)	-11.50; 55,404.92	0.08 (1.25)	-6.00; 5.00	0.08 (1.24)	-6.00; 5.00
Number of measurements	4.68 (4.08)	2; 145	4.46 (4.00)	1; 113	4.59 (4.02)	2; 113
**Girls**						
Age (months)	31.76 (16.51)	0.00; 59.00	31.86 (16.50)	0.00; 59.00	31.84 (16.50)	0.00; 59.00
Length/height (cm)	126.70 (5325.19)	0.00; 990,000.00	89.40 (14.66)	45.00; 120.00	89.38 (14.66)	45.00; 120.00
L/HAZ	11.50 (1681.45)	-29.10; 381,301.70	-0.29 (1.49)	-6.00; 6.00	-0.29 (1.49)	-6.00; 6.00
Number of measurements	4.63 (3.98)	2; 88	4.39 (3.90)	1; 88	4.53 (3.92)	2; 88
Age (months)	31.76 (16.51)	0.00; 59.00	31.80 (16.50)	0.00; 59.00	31.77 (16.50)	0.00; 59.00
Weight (kg)	15.07 (143.34)	0.00; 263,642.00	13.24 (4.09)	1.70; 36.80	13.23 (4.08)	1.70; 36.80
WAZ	0.89 (53.86)	-11.32; 65,928.75	0.09 (1.20)	-6.00; 5.00	0.09 (1.20)	-6.00; 5.00
Number of measurements	4.63 (3.98)	2; 88	4.42 (3.91)	1; 88	4.54 (3.92)	2; 88

In boys, the percentage of LOs varied from 0.02% (<-6 / >+6) to 1.47% (<-3 / >+3) for length/height and from 0.07 to 1.44% for weight, respectively. In girls, the percentage of LOs varied from 0.01 to 1.50% for length/height and from 0.08 to 1.45% for weight. There were more positive LOs for length/height and weight measurements, except for length/height with the cutoff value of $-6/+6$ (Fig. 1). After removing LOs, descriptive statistics were similar across the different cutoffs used to flag the LOs and to the previous statistics obtained after the removal of POs only (Table 2).

**Table 2:** Descriptive statistics for anthropometric variables after removing longitudinal outliers according to different cutoffs. Brazilian Food and Nutrition Surveillance System (SISVAN), 2008–2017

	Dataset after removing LOs according to different cutoffs							
	< -3 / > +3		< -4 / > +4		< -5 / > +5		< -6 / > +6	
	Mean (SD)	Min; Max	Mean (SD)	Min; Max	Mean (SD)	Min; Max	Mean (SD)	Min; Max
**Boys**								
Age (months)	31.37 (16.57)	0.00; 59;00	31.42 (16.55)	0.00; 59.00	31.44 (16.54)	0.00; 59.00	31.45 (16.53)	0.00; 59.00
Length/height (cm)	89.90 (14.48)	45.00; 120.00	89.98 (14.51)	45.00; 120.00	90.01 (14.53)	45.00; 120.00	90.02 (14.53)	45.00; 120.00
L/HAZ	-0.38 (1.45)	-6.00; 6.00	-0.37 (1.51)	-6.00; 6.00	-0.37 (1.53)	-6.00; 6.00	-0.36 (1.53)	-6.00; 6.00
Number of measurements	4.52 (3.99)	1; 113	4.57 (4.00)	1; 113	4.58 (4.01)	1; 113	4.58 (4.01)	1; 113
Age (months)	31.24 (16.53)	0.00; 59.00	31.32 (16.53)	0.00; 59.00	31.36 (16.53)	0.00; 59.00	31.37 (16.53)	0.00; 59.00
Weight (kg)	13.52 (3.93)	1.70; 34.60	13.56 (3.97)	1.70; 34.60	13.58 (4.00)	1.70; 34.60	13.58 (4.01)	1.70; 34.60
WAZ	0.06 (1.20)	-6.00; 5.00	0.07 (1.22)	-6.00; 5.00	0.08 (1.23)	-6.00; 5.00	0.08 (1.24)	-6.00; 5.00
Number of measurements	4.54 (3.99)	1; 113	4.57 (4.00)	1; 113	4.58 (4.01)	1; 113	4.59 (4.02)	1; 113
**Girls**								
Age (months)	31.76 (16.54)	0.00; 59.00	31.81 (16.51)	0.00; 59.00	31.83 (16.50)	0.00; 59.00	31.83 (16.50)	0.00; 59.00
Length/height (cm)	89.26 (14.60)	45.00; 120.00	89.34 (14.64)	45.00; 120.00	89.37 (14.65)	45.00; 120.00	89.38 (14.66)	45.00; 120.00
L/HAZ	-0.31 (1.40)	-6.00; 6.00	-0.30 (1.46)	-6.00; 6.00	-0.29 (1.56)	-6.00; 6.00	-0.29 (1.49)	-6.00; 6.00
Number of measurements	4.47 (3.89)	1; 88	4.52 (3.91)	1; 88	4.53 (3.91)	1; 88	4.53 (3.92)	1; 88
Age (months)	31.63 (16.49)	0.00; 59.00	31.71 (16.49)	0.00; 59.00	31.75 (16.50)	0.00; 59.00	31.76 (16.50)	0.00; 59.00
Weight (kg)	13.14 (3.97)	1.70; 35.80	13.19 (4.02)	1.70; 36.80	13.21 (4.05)	1.70; 36.80	13.22 (4.07)	1.70; 36.80
WAZ	0.06 (1.16)	-6.00; 5.00	0.08 (1.18)	-6.00; 5.00	0.08 (1.19)	-6.00; 5.00	0.09 (1.20)	-6.00; 5.00
Number of measurements	4.49 (3.89)	1; 88	4.59 (3.95)	1; 88	4.53 (3.92)	1; 88	4.54 (3.92)	1; 88

Notes: LOs: longitudinal outliers, SD: standard deviation, L/HAZ: length/height-for-age z-score, WAZ: weight-for-age z-score

**Fig. 2:**
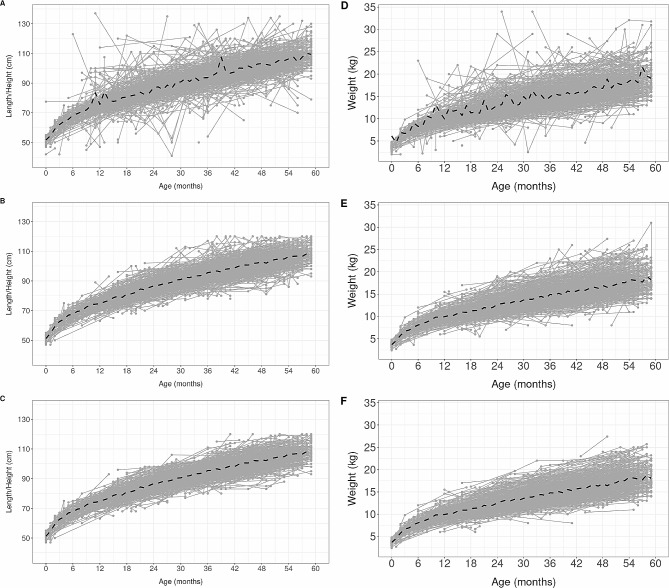
Trajectories of length/height (**A**, **B**, **C**) and weight (**D**, **E**, **F**) of 1,000 randomly selected boys in the initial dataset (**A** and **D**) and after removing POs (**B** and **E**) and LOs (**C** and **F**). Notes: Longitudinal outliers (LOs) were based on the most conservative cutoff (<-3 / >+3). The black dashed line represents the observed mean curve based on all observations. To maintain scale and comparability there were observations that were not shown in panels A and D (available upon request)

**Fig. 3:**
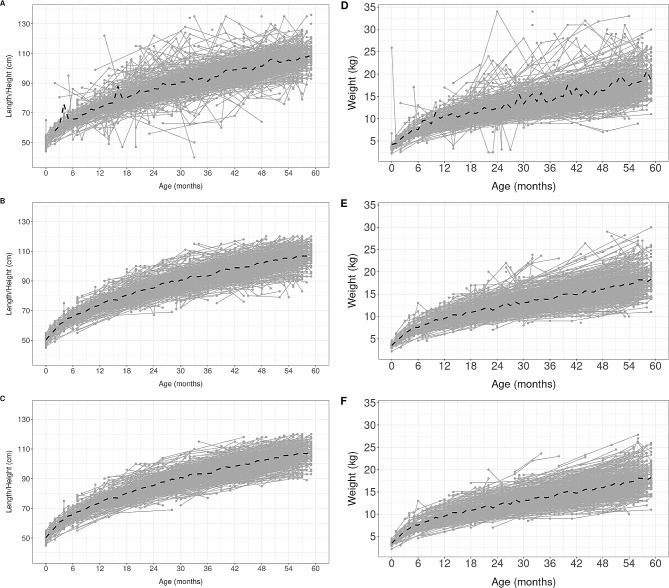
Trajectories of length/height (**A**, **B**, **C**) and weight (**D**, **E**, **F**) of 1,000 randomly selected girls in the initial dataset (**A** and **D**) and after removing POs (**B** and **E**) and LOs (**C** and **F**). Notes: Longitudinal outliers (LOs) were based on the most conservative cutoff (<-3 / >+3). The black dashed line represents the observed mean curve based on all observations. To maintain scale and comparability there were observations that were not shown in panels A and D (available upon request)

The trajectories of length/height and weight of 1,000 randomly selected boys and 1,000 randomly selected girls in the initial dataset showed mean curves with peaks in both sexes (Figs. 2A and D and 3A and D). Most substantial changes in the trajectories of those randomly selected boys and girls occurred with the removal of POs, where the peaks were absent in the mean observed curves (Figs. 2B and E and 3B and E). Only a few changes were observed in both sexes’ trajectories and mean curves when the LOs were removed, regardless of the cutoff value (Figs. 2C and F and 3C and F, S1, S2, S3, and S4).

The prevalence of child growth indicators presented changes when POs were removed. Among the extreme categories of HAZ and WAZ, the prevalence of severe stunting (HAZ<-3), severe underweight (WAZ < -3) and overweight (WAZ > 2) were the most affected by the removal of POs. Concerning the initial dataset, the prevalence of severe stunting decreased from 4.75 to 4.36% for boys (-0.39%) and from 3.91 to 3.56% for girls (-0.35%) after removing POs only. The prevalence of overweight for age (WAZ > 2) decreased from 6.94 to 6.15% for boys (-0.79%) and from 6.34 to 5.62% for girls (-0.72%). To a lesser extent, these categories were also affected by the removal of LOs, especially for the most restrictive cutoff (<-3/ >+3) (Tables 3 and 4).

**Table 3:** Prevalence of child growth indicators in the initial dataset and after removing population and longitudinal outliers according to different cutoffs - **boys**. Brazilian Food and Nutrition Surveillance System (SISVAN), 2008–2017

	Initial dataset	Dataset after removing POs	Dataset after removing children with only one measurement	Dataset after removing LOs according to different cutoffs			
				< -3/ > +3	< -4/ > +4	< -5/ > +5	< -6/ > +6
Severe stunting	4.75	4.36	4.32	3.87	4.19	4.29	4.32
Moderate stunting	7.70	7.93	7.92	7.98	7.95	7.93	7.93
Adequate	84.60	87.71	87.75	88.15	87.85	87.78	87.76
BIV	2.89	-	-	-	-	-	-
Missing	0.06	-	-	-	-	-	-
Severe underweight	1.17	0.99	0.98	0.82	0.91	0.95	0.97
Moderate underweight	3.19	3.19	3.19	3.11	3.17	3.18	3.19
Adequate	86.91	89.66	84.79	90.73	90.16	89.90	89.90
Overweight	6.94	6.15	6.09	5.34	5.76	5.96	6.04
BIV	1.63	-	-	-	-	-	-
Missing	0.16	-	-	-	-	-	-

Notes: POs: population outliers, LOs: longitudinal outliers, L/HAZ: length/height-for-age z-score, WAZ: weight-for-age z-score

**Table 4:** Prevalence of child growth indicators in the initial dataset and after removing population and longitudinal outliers according to different cutoffs – **girls**. Brazilian Food and Nutrition Surveillance System (SISVAN), 2008–2017

	Initial dataset	Dataset after removing POs	Dataset after removing children with only one measurement	Dataset after removing LOs according to different cutoffs			
				< -3/ > +3	< -4/ > +4	< -5/ > +5	< -6/ > +6
Severe stunting	3.91	3.56	3.53	3.04	3.39	3.50	3.52
Moderate stunting	6.68	6.87	6.86	6.91	6.89	6.87	6.86
Adequate	85.51	89.57	89.61	90.05	89.72	89.63	89.61
BIV	2.83	-	-	-	-	-	-
Missing	0.07	-	-	-	-	-	-
Severe underweight	0.97	0.83	0.82	0.70	0.77	0.81	0.82
Moderate underweight	2.90	2.91	2.90	2.84	2.89	2.90	2.90
Adequate	88.06	90.64	90.70	91.70	91.15	90.89	90.78
Overweight	6.34	5.62	5.57	4.76	5.19	5.40	5.50
BIV	1.52	-	-	-	-	-	-
Missing	0.21	-	-	-	-	-	-

Notes: POs: population outliers, LOs: longitudinal outliers, L/HAZ: length/height-for-age z-score, WAZ: weight-for-age z-score

After removing the POs, there were 1,514,066 (6.58%) “decreasing heights” observations for boys and 1,579,379 (6.63%) for girls (data not shown). Unlike the model-based residual approach used to flag and remove LOs, removing “decreasing heights” seemed to change the L/HAZ means and prevalence estimates (Table S2). Regarding the dataset after removing POs (and removing children with a single measurement), the L/HAZ mean increased from − 0.36 to -0.29 for boys (+ 0.07) and − 0.29 to -0.21 for girls (+ 0.08), while the prevalence of severe stunting decreased from 4.32 to 3.39% in boys (-0.93%) and 3.53–2.62% in girls (-0.91%) after removing “decreasing heights” (see Tables S3 and S4). Additional results enable comparison of prevalences of child growth indicators based on HAZ and WAZ, for boys and girls, at each data cleaning process. These results are separate for length/height (with and without the presence of “decreasing heights”) and weight (see Tables S3, S4, and S5).

The proportion of POs and LOs (based on the cutoff value of -3/+3) removed at each age was calculated. There were more observations classified as POs in younger ages in comparison to those classified as LOs (Figure S5).

## Discussion

In this study, we adapted and tested methods to identify BIVs in a large administrative dataset with longitudinal anthropometric measurements of children from 0 to 59 years old. Using WHO cutoffs for BIVs, we identified POs in 5.74% and 5.31% of the length/height and 5.19% and 4.74% of the weight measurements, in boys and girls respectively. After removing POs, different cutoffs were used to flag LOs based on the residuals of linear ME models. The maximum percentage of LOs was 1.50% considering both sexes, even when a more restricted cutoff (<-3/>+3) was applied. In general, the removal of POs played the largest role in the child growth trajectories and in the estimates of the prevalence of child growth indicators. In contrast, the subsequent removal of LOs had lower impact on subjects’ trajectories, regardless of the cutoff used to flag those measurements. This can be expected since POs are removed first and therefore LOs account for an additional cleaning in the dataset. In addition, POs and LOs are unequally distributed according to age. This behavior may be a reflection of using only a random intercept in the mixed-effects model. Alternatively, measurements are also unequally distributed along the age, often with a lower quantity at the beginning and end of the follow-up period. Another point to consider is that the proportion of LOs tends to increase mainly in the months when POs were less prevalent, considering both length/height and weight. Removing the “decreasing heights” substantially influenced child growth outcomes using L/HAZ measurements. As an example, prevalence of severe stunting decreased 21.53% (from 4.32 to 3.39%) in boys and 25.78% (from 3.53 to 2.62%) in girls.

Reference charts and the sample’s distributions are still widely used methods to flag and clean BIVs in anthropometric data, especially in cross-sectional studies [6]. The percentage of POs in our dataset exceeded 1% for both L/HAZ and WAZ, suggesting poor data quality according to the WHO implausibility system [10]. Even child growth data collected primarily for research purposes often fail to meet these quality criteria, mainly due to the difficulty of performing anthropometric assessment in young children and inadequate facility conditions in low-resource areas [4, 5]. This situation underscores the importance of correctly identifying BIVs in the datasets from those locations.

Recent studies have proposed different methods to identify LOs based on growth trajectories of length/height and weight in children, including model-based approaches using conditional growth percentiles [7], jackknife residuals from linear regression [9], studentized residuals from mixed-effects models [8], Cook’s distance, DFFITS and DFBETAS [17]. To the best of our knowledge, the method based on studentized residuals from ME models [8] is the most feasible and appropriate, since it is not computationally expensive and accommodates the nonlinearity in child growth patterns and the substantial variability in the number and spacing of anthropometric measures and age. However, it may present limitations in a large data base such as SISVAN. Therefore, we used ME approach and scaled residuals - and tested four cutoffs from more conservative (-3/+3) to more flexible (-6/+6) ranges to flag LOs. The percentages of LOs varied across the cutoffs as expected, and always comprised at most 1.5% of the measurements, even using the more conservative cutoff. Thus, removing LOs based on this approach, after the removal of POs, presented lower impact on the child growth trajectories and the prevalence of indicators, regardless of the cutoff adopted.

A previous study using a similar method to flag LOs found a negligible impact of removing LOs (-6/+6) on the estimated prevalence of child BMI categories [8]. Unlike our study, POs and LOs represented less than 0.3% of their dataset. However, the authors also observed that the exclusion of POs resulted in smaller changes in the mean weight, height, and BMI over time compared with the exclusion of LOs. In our study, the prevalence of severe stunting and overweight for age dropped approximately 0.39% and 0.79% for boys and 0.35% and 0.72% for girls, after removing POs, respectively. These values represented a relative difference in the removal of POs in relation to the initial step of the cleaning process of 13.62% and 13.99% for severe stunting, and of 16.09% and 15.80% for overweight, for boys and girls respectively.

We studied the influence of biologically implausible decreases in height measurements (“decreasing heights”). This feature represented 6.63% of the observations, and removing those measurements substantially changed child growth outcomes compared to LOs. After excluding POs and children with a single measurement, the prevalence of severe stunting decreased by 0.93% and 0.91% for boys and girls, respectively, due to the removal of “decreasing heights”. A recent study developed an automated protocol for cleaning pediatric height and weight from longitudinal electronic health records [17]. In this study, inflated error rates for height measurements were also detected due to small but physiologically implausible decreases in height exceeding − 2 cm. It is essential to highlight that to detect the “decreasing heights”, the first measurement is assumed to be correct. Thus, this method can fail to detect outliers if the first measurement is implausible.

Although removing BIVs is important, especially in datasets in which data collection is not standardized, such as the SISVAN, caution must be taken. The removal of extremely high values from the tails of the distribution may introduce unknown biases and limit the accurate estimation of prevalence and growth trajectories of children at increased risk of malnutrition [18, 19]. In the current study, the higher proportion of POs in the positive part of the interval, especially for weight (WAZ > + 5), could suggest that these are, in fact, values from children who are heavier and exceed 5 standard deviations of the WHO reference charts.

In addition, the lack of a standard cutoff to define LOs based on regression residuals remains a concern. The decision of the best cutoff should be study-based and consider that some inaccurate values that may not belong to a child series of observations will be kept in the dataset if we use cutoffs with larger ranges. On the other hand, extreme but true values can be unnecessarily removed if we use more restrictive cutoffs with lower ranges. These are important reflections to be made when flagging and removing BIVs.

Our study is the first to use real-world ‘big data’ of longitudinal anthropometric measurements to detect and evaluate the impact of different types of outliers. The SISVAN dataset, although not representative of the Brazilian population, is an important data source for the Ministry of Health to monitor nutritional indicators throughout the life course and includes data collected in the largest public healthcare system worldwide. Thus, correctly identifying and removing BIVs in this dataset can change the estimates of the prevalence of child growth indicators extracted from the SISVAN. Besides, it can also affect the current public healthcare policies that are often made based on these results. We observed a lack of information in some studies in the field regarding the residuals used to flag LOs. Our study presented the formula used to calculate the scaled residuals and confirmed that the statistical software calculated them using the same formula. Since there are different types of regression residuals, future studies should describe this step in more detail for clarity and research reproducibility.

A major limitation for our study and others in the field is the lack of a gold standard of what are the ‘true’ BIVs. This means we could only compare the number of observations flagged as POs and LOs by different methods but could not determine whether both were accurate. Future studies using data collected through standardized approaches and simulation techniques could help in comparing methods. More recently, machine learning has been proposed as a promising technique for improving outlier detection in small sample [20]. Thus, future studies using big datasets could also consider artificial intelligence methods. In addition, mixed-effect models used to detect LOs included only random intercept due to the sample size. It is suggested that, if possible, to fit the model with both random intercept and slope.

## Conclusions

The approach used in this study can be a tool to identify systematically implausible length/height and weight measurements from routinely collected data of children in primary healthcare services. This tool examines POs and LOs. Although both methods were able to detect BIVs in our dataset, the initial removal of POs played the largest role in the child growth trajectories and in the prevalence estimates of child growth, as this is the first step in the data cleaning process. In contrast, the subsequent removal of LOs had lower impact on subjects’ trajectories, regardless of the cutoff used to flag those measurements. Nonetheless, this latter approach was simple and computationally reasonable, and it can be particularly useful when integrated with an approach to flag POs, to detect and remove outliers in big longitudinal datasets before estimating growth trajectories.

It is also important to mention that the sequence presented here is a proposal to deal with outliers with large administrative longitudinal data. The decision regarding the appropriate approach is data-dependent and should be discussed by the team of researchers. Factors such as the average number of measurements per individual, the percentage of individuals with many repeated measurements, and the amount of data flagged by each criterion should be considered in this decision.

### Electronic supplementary material

Below is the link to the electronic supplementary material.

Supplementary Material 1

## Data Availability

The data that support the findings of this study are available upon request available from the Brazilian Ministry of Health, but restrictions apply to the availability of these data, which were used under license for the current study, and so are not publicly available. Data are however available upon request to the Ministry of Health, following specific Brazilian laws. The codes used in this analysis are also available upon request to the corresponding author (rcrsilva@ufba.br).

## Abbreviations

BIV: Biologically implausible value
BMIZ: Body-mass-index-for-age z-score
CIDACS: Centre for Data and Knowledge Integration for Health
L/HAZ: Length/height-for-age z-score
LOs: Longitudinal outliers
POs: Population outliers
SISVAN: Brazilian Food and Nutrition Surveillance System
WAZ: Weight-for-age z-score
WHO: World Health Organization
WHZ: Weight-for-length/height z-score
