# Supplementary material for: Identifying biologically implausible values in big longitudinal data: an example applied to child growth data from the Brazilian food and nutrition surveillance system
Source: BMC Med Res Methodol. 2024 Feb 15;24:38. doi: 10.1186/s12874-024-02161-1 (PMC10868032; doi:10.1186/s12874-024-02161-1)
Supplement: Supplementary file 1 — Supplementary Material 1 [file 12874_2024_2161_MOESM1_ESM.docx]

**Identifying biologically implausible values in big longitudinal data: an example applied to child growth data from the Brazilian Food and Nutrition Surveillance System**

**SUPPLEMENTARY MATERIAL**

**Figure S1**: Trajectories of length/height of 1000 randomly selected boys.

**Figure S2**: Trajectories of length/height of 1000 randomly selected girls.

**Figure S3**: Trajectories of weight of 1000 randomly selected boys.

**Figure S4**: Trajectories of weight of 1000 randomly selected girls.

**Figure S5**: Proportion of both POs and LOs (cutoff value of -3/+3) removed at each age.

**Table S1**: Frequency (and percentage) of observations classified as LOs based on different types of residuals and software estimations and according to cut-off values.

**Table S2**: Descriptive statistics for age, length/height, L/HAZ, and number of measurements in the initial dataset, after removing population outliers, removing children with only one measurement, and “decreasing heights”. Brazilian Food and Nutrition Surveillance System (SISVAN), 2008-2017.

**Table S3:** Prevalence of child growth indicator (L/HAZ) in the initial dataset, after removing population outliers, after removing children with only one measurement, and longitudinal outliers according to different cutoffs – for **height** **with decreasing height in dataset**. Brazilian Food and Nutrition Surveillance System (SISVAN), 2008–2017.

**Table S4:** Prevalence of child growth indicator (L/HAZ) in the initial dataset, after removing population outliers, after removing children with only one measurement, and longitudinal outliers according to different cutoffs – for **height** **without decreasing height in dataset**. Brazilian Food and Nutrition Surveillance System (SISVAN), 2008–2017.

**Table S5:** Prevalence of child growth indicator (WAZ) in the initial dataset, after removing population outliers, after removing children with only one measurement, and longitudinal outliers according to different cutoffs – for **weight**. Brazilian Food and Nutrition Surveillance System (SISVAN), 2008–2017.


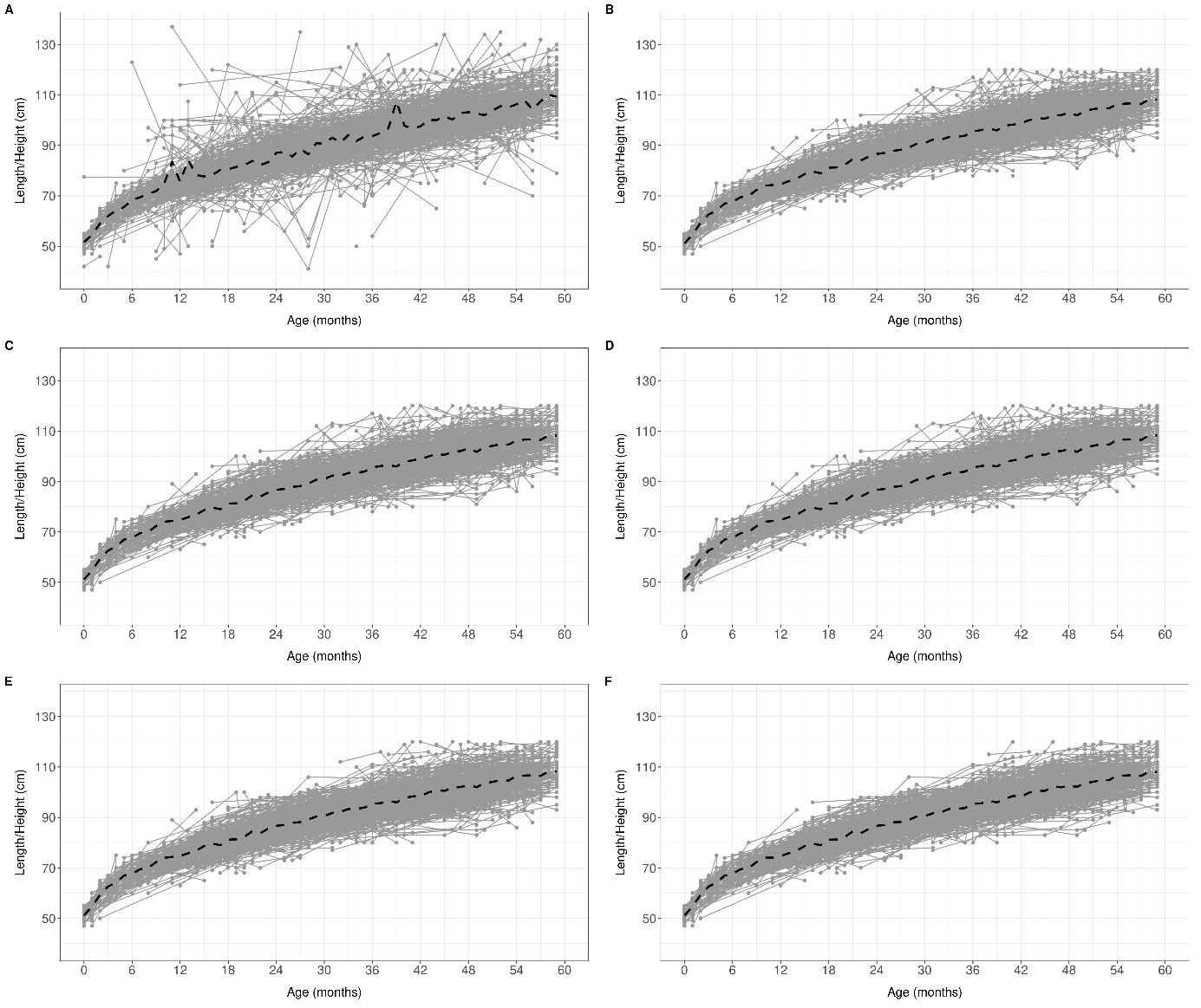


**Figure S1**: Trajectories of length/height of 1000 randomly selected boys.

(A) Initial dataset, (B) after removing POs, (C) after removing LOs cutoff -6/+ 6, (D) after removing LOs cutoff -5/+ 5, (E) after removing LOs cutoff -4/+ 4, (F) after removing LOs cutoff -3/+ 3.

Note: black dashed line is the observed mean curve based on all observations in the step.


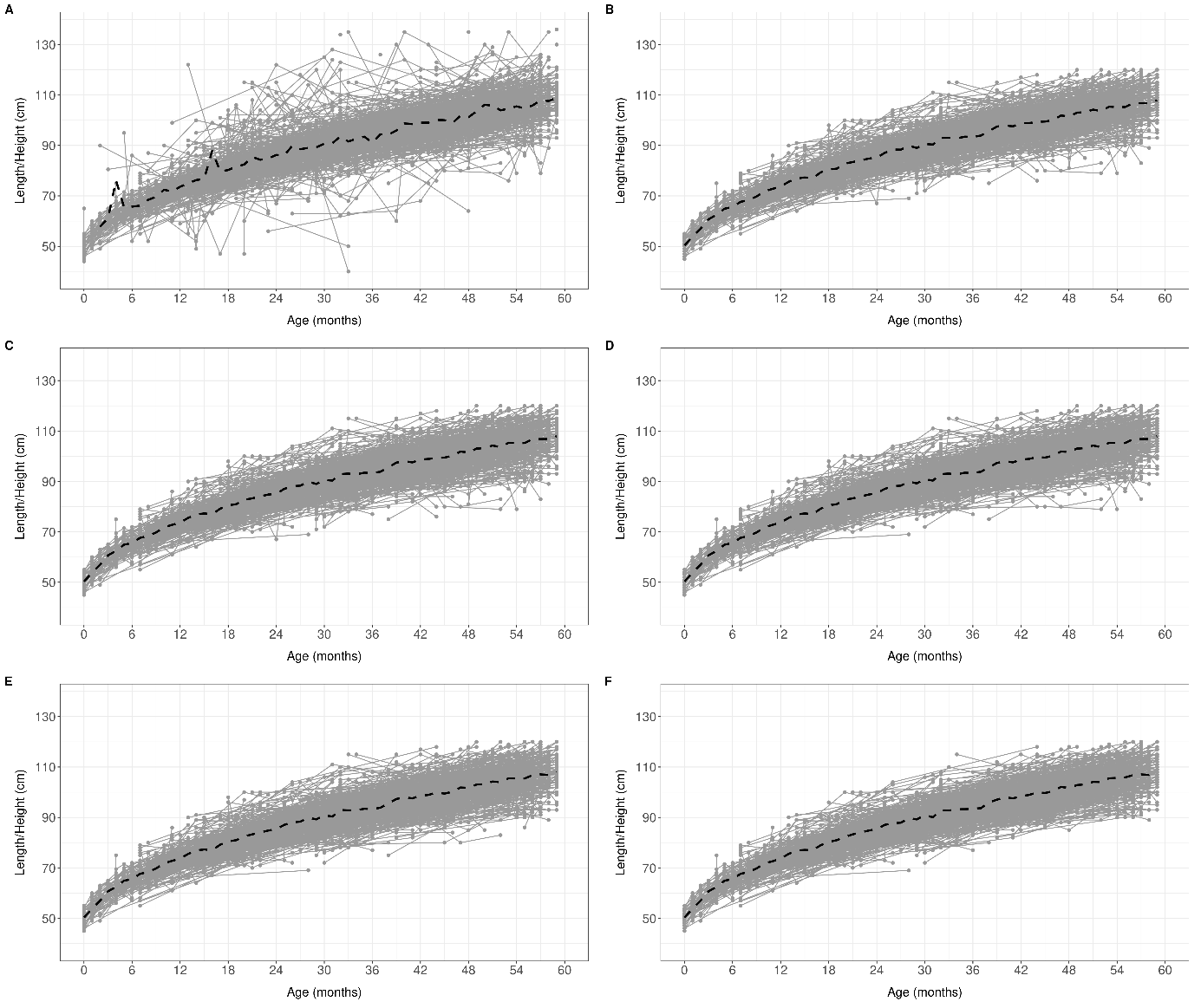


**Figure S2**: Trajectories of length/height of 1000 randomly selected girls.

(A) Initial dataset, (B) after removing POs, (C) after removing LOs cutoff -6/+ 6, (D) after removing LOs cutoff -5/+ 5, (E) after removing LOs cutoff -4/+ 4, (F) after removing LOs cutoff -3/+ 3.

Note: black dashed line is the observed mean curve based on all observations in the step.


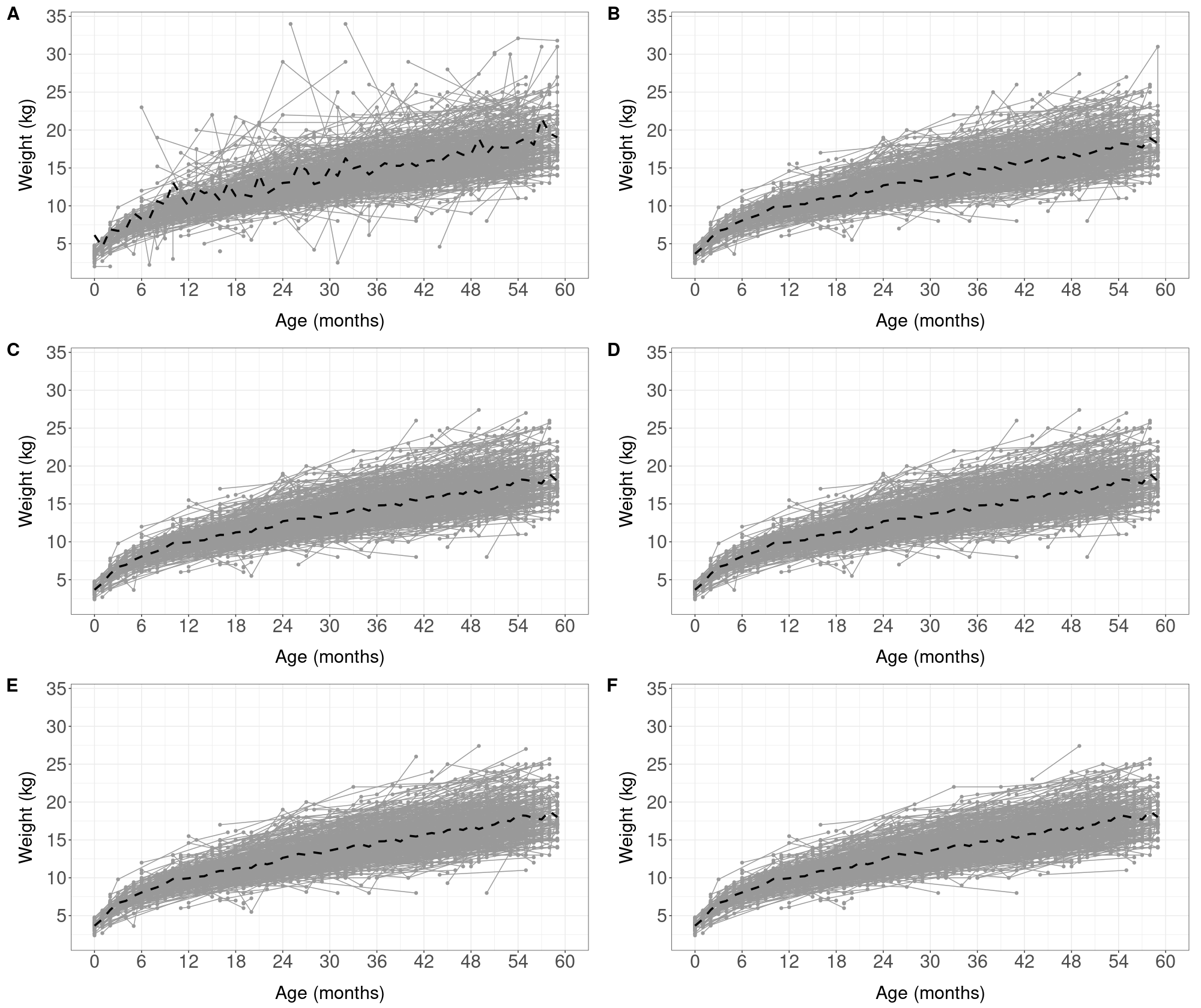


**Figure S3**: Trajectories of weight of 1000 randomly selected boys.

(A) Initial dataset, (B) after removing POs, (C) after removing LOs cutoff -6/+ 6, (D) after removing LOs cutoff -5/+ 5, (E) after removing LOs cutoff -4/+ 4, (F) after removing LOs cutoff -3/+ 3.

Note: black dashed line is the observed mean curve based on all observations in the step.


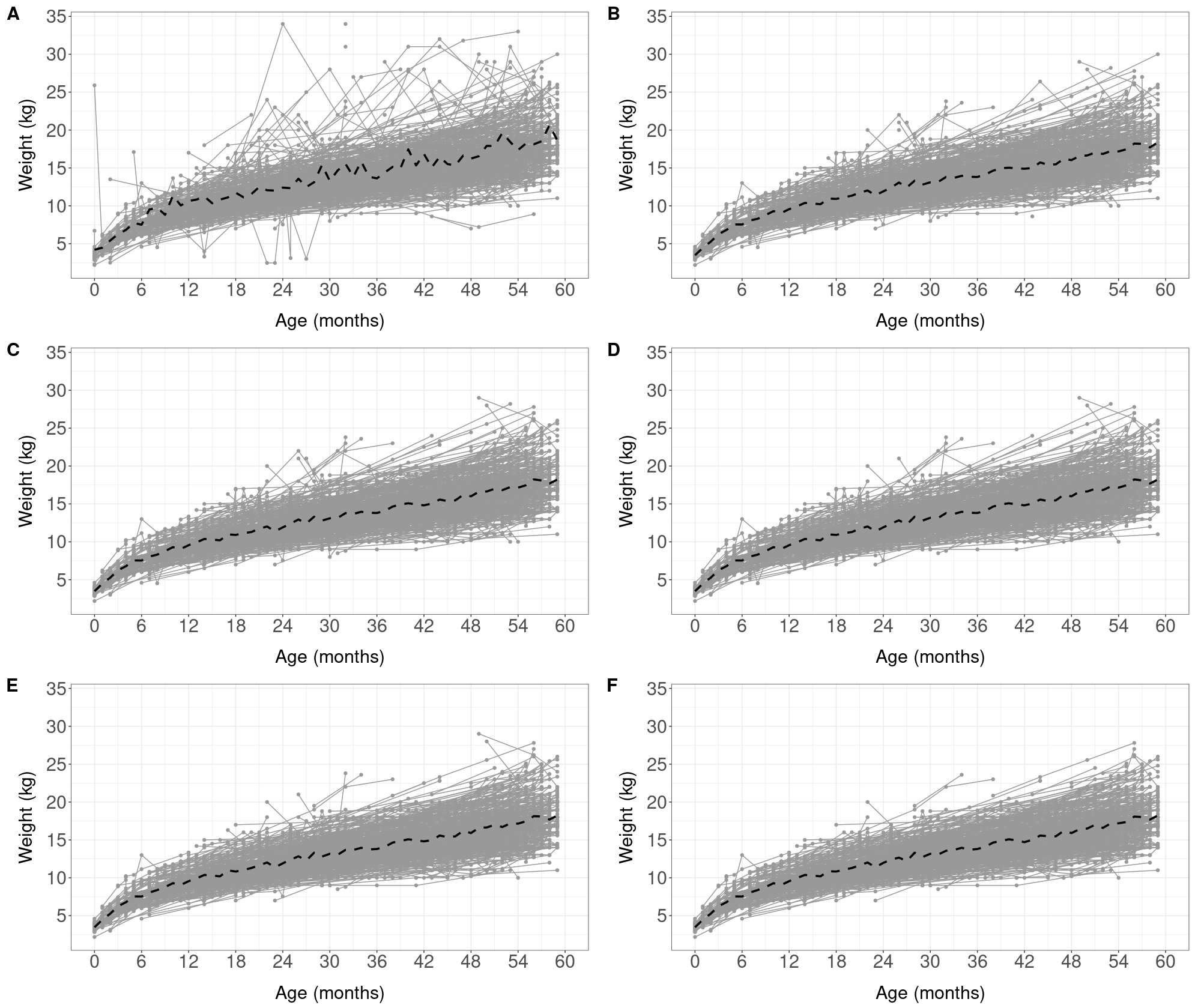


**Figure S4**: Trajectories of weight of 1000 randomly selected girls.

(A) Initial dataset, (B) after removing POs, (C) after removing LOs cutoff -6/+ 6, (D) after removing LOs cutoff -5/+ 5, (E) after removing LOs cutoff -4/+ 4, (F) after removing LOs cutoff -3/+ 3.

Note: black dashed line is the observed mean curve based on all observations in the step.

**Figure S5**: Proportion of both POs and LOs (cutoff value of -3/+3) removed at each age, for length/height (a) and weight (b).

| 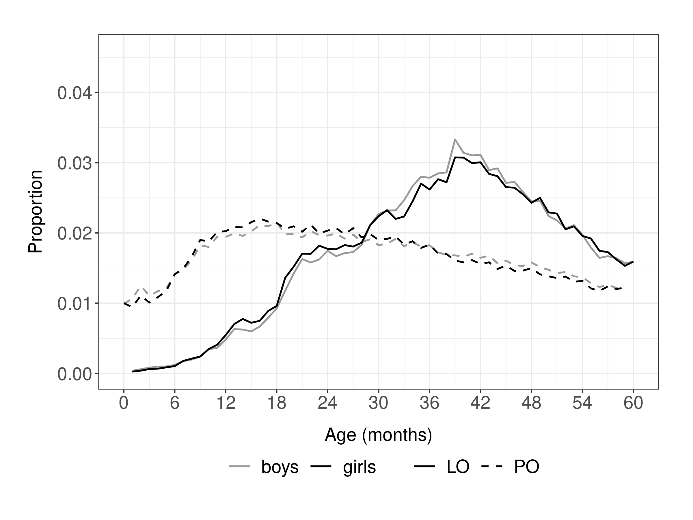 | 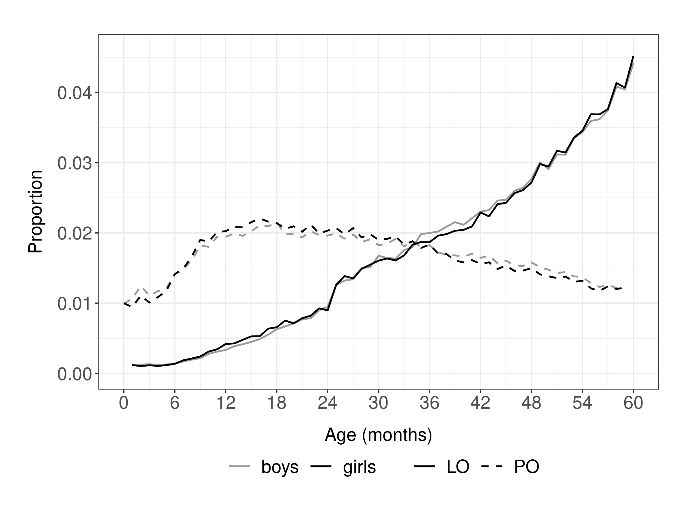 |
| --- | --- |
| 1. Height (cm) | 1. Weight (kg) |

**Table S1:** Frequency (and percentage) of observations classified as LOs based on different types of residuals and software estimations and according to cut-off values.

|  | < -3 / > +3 | < -4 / > +4 | < -5 / > +5 | < -6 / > +6 |
| --- | --- | --- | --- | --- |
| Scaled | 72 | 35 | 15 | 5 |
|  | (1.61%) | (0.78%) | (0.33%) | (0.11%) |
| Studentized  (redres) | 96 | 48 | 25 | 10 |
|  | (2.14%) | (1.07%) | (0.58%) | (0.22%) |
| Studentized  (R-base) | 96 | 48 | 25 | 10 |
|  | (2.14%) | (1.07%) | (0.58%) | (0.22%) |
| Studentized  (Stata) | 68 | 29 | 16 | 4 |
|  | (1.52%) | (0.65%) | (0.36%) | (0.09%) |

Note: Table S1 shows a very brief comparison of different residuals and software estimations to identifying LO. The comparison was performed for height measurements of boys only, as there is no suspicion that the other anthropometric measure (weight) or sex (girls) would behave differently. In addition, it was based on a sample of 1000 randomly selected boys. The alternatives could be in software R: (i) scaled residuals, (ii) studentized residuals via redres package, (iii) studentized residuals via R-base. Or, they could be in software Stata, (iv) studentized residuals. This comparison was performed to confirm validation of scaled residuals, since it is very computational costly to compute studentized residuals when it runs.

**Table S2**: Descriptive statistics for age, length/height, L/HAZ, and number of measurements in the initial dataset and after removing population outliers and “decreasing heights”. Brazil’s Food and Nutrition Surveillance System, 2008-2017.

|  | **Initial dataset** | | **Dataset after**  **removing POs** | | **Dataset after removing children with only one measurement** | | **Dataset after removing “decreasing heights”** | | **Dataset after removing children with only one measurement** | |
| --- | --- | --- | --- | --- | --- | --- | --- | --- | --- | --- |
|  | Mean  (SD) | Min ; Max | Mean (SD) | Min ; Max | Mean (SD) | Min ; Max | Mean (SD) | Min ; Max | Mean (SD) | Min ; Max |
| Boys |  |  |  |  |  |  |  |  |  | |
| Age (months) | 31.41  (16.55) | 0.00; 59.00 | 31.47 (16.54) | 0.00; 59.00 | 31.45 (16.53) | 0.00; 59.00 | 31.03 (16.58) | 0.00 ; 59.00 | 30.99 (16.59) | 0.00 ; 59.00 |
| Length/height (cm) | 136.40 (5914.07) | 0.00; 993,900.00 | 90.04 (14.54) | 45.00; 120.00 | 90.02 (14.53) | 45.00; 120.00 | 89.96 (14.70) | 45.00 ; 120.00 | 89.90 (14.69) | 45.00 ; 120.00 |
| L/HAZ | 15.40 (2034.79) | -32.10; 433,915.30 | -0.36 (1.54) | -6.00; 6.00 | -0.36 (1.53) | -6.00; 6.00 | -0.29 (1.50) | -6.00 ; 6.00 | -0.30 (1.49) | -6.00 ; 6.00 |
| Number of  Measurements | 4.68  (4.08) | 2;145 | 4.43 (3.99) | 1; 113 | 4.58 (4.01) | 2; 113 | 4.28 (3.75) | 1 ; 113 | 4.37 (3.76) | 2; 113 |
| Girls |  |  |  |  |  |  |  |  |  | |
| Age (months) | 31.76  (16.51) | 0.00; 59.00 | 31.86 (16.50) | 0.00; 59.00 | 31.84 (16.50) | 0.00; 59.00 | 31.42  (16.55) | 0.00 ; 59.00 | 31.38 (16.56) | 0.00 ; 59.00 |
| Length/height (cm) | 126.70  (5325.19) | 0.00; 990,000.00 | 89.40 (14.66) | 45.00; 120.00 | 89.38 (14.66) | 45.00; 120.00 | 89.33  (14.83) | 45.00 ; 120.00 | 89.27 (14.82) | 45.00 ; 120.00 |
| L/HAZ | 11.50  (1681.45) | -29.10; 381,301.70 | -0.29 (1.49) | -6.00; 6.00 | -0.29 (1.49) | -6.00; 6.00 | -0.21  (1.45) | -6.00 ; 6.00 | -0.22 (1.44) | -6.00 ; 6.00 |
| Number of  Measurements | 4.63  (3.98) | 2; 88 | 4.39 (3.90) | 1; 88 | 4.53 (3.92) | 2; 88 | 4.23  (3.66) | 1 ; 88 | 4.32 (3.67) | 2 ; 88 |

POs: population outliers, SD: standard deviation, L/HAZ: length/height-for-age z-score, WAZ: weight-for-age z-score.

**Table S3:** Prevalence of child growth indicator (L/HAZ) in the initial dataset, after removing population outliers, after removing children with only one measurement, and longitudinal outliers according to different cutoffs – for **height** **with decreasing height in dataset**. Brazilian Food and Nutrition Surveillance System (SISVAN), 2008–2017.

|  | **Initial**  **dataset** | **%** | **Dataset after**  **removing POs** | **%** | **Dataset after**  **removing**  **children with**  **only one**  **measurement** | **%** | **Dataset after removing LOs according to different cutoffs** | | | | | | | |
| --- | --- | --- | --- | --- | --- | --- | --- | --- | --- | --- | --- | --- | --- | --- |
|  |  |  |  |  |  |  | <-3/ >+3 | % | <-4 / >+4 | % | <-5 / >+5 | % | <-6 / >+6 | % |
| Boys |  |  |  |  |  |  |  |  |  |  |  |  |  |  |
| Severe  Stunting | 1,172,216 | 4.75 | 1,012,549 | 4.36 | 994,630 | 4.32 | 876,387 | 3.87 | 961,179 | 4.19 | 986,995 | 4.29 | 993,230 | 4.32 |
| Moderate  Stunting | 1,901,603 | 7.70 | 1,842,482 | 7.93 | 1,822,941 | 7.92 | 1,809,683 | 7.98 | 1,822,100 | 7.95 | 1,822,909 | 7.93 | 1,822,941 | 7.93 |
| Adequate | 20,895,596 | 84.60 | 20,367,350 | 87.71 | 20,186,050 | 87.75 | 19,981,215 | 88.15 | 20,130,245 | 87.85 | 20,175,939 | 87.78 | 20,184,901 | 87.76 |
| BIV | 714,122 | 2.89 | - |  | - |  | - |  | - | - | - | - | - | - |
| Missing | 15,094 | 0.06 | - | - | - | - | - | - | - | - | - | - | - | - |
| Total | 24,698,631 | 100.00 | 23,222,381 | 100.00 | 23,003,621 | 100.00 | 22,667,285 | 100.00 | 22,913,524 | 100.00 | 22,985,843 | 100.00 | 23,001,072 | 100.00 |
| Girls |  |  |  |  |  |  |  |  |  |  |  |  |  |  |
| Severe  Stunting | 994,500 | 3.91 | 855,414 | 3.56 | 841,128 | 3.53 | 713,488 | 3.04 | 804,673 | 3.39 | 833,215 | 3.50 | 839,914 | 3.52 |
| Moderate  Stunting | 1,701,572 | 6.68 | 1,652,065 | 6.87 | 1,635,587 | 6.86 | 1,622,764 | 6.91 | 1,634,812 | 6.89 | 1,635,553 | 6.87 | 1,635,586 | 6.86 |
| Adequate | 22,021,756 | 86.51 | 21,535,505 | 89.57 | 21,357,593 | 89.61 | 21,140,327 | 90.05 | 21,299,816 | 89.72 | 21,347,647 | 89.63 | 21,356,632 | 89.61 |
| BIV | 720,867 | 2.83 | - | - | - | - | - |  | - | - | - | - | - | - |
| Missing | 17,412 | 0.07 | - | - | - | - | - | - | - | - | - | - | - | - |
| Total | 25,456,107 | 100.00 | 24,042,984 | 100.00 | 23,834,308 | 100.00 | 23,476,579 | 100.00 | 23,739,301 | 100.00 | 23,816,415 | 100.00 | 23,832,132 | 100.00 |

**Table S4:** Prevalence of child growth indicator (L/HAZ) in the initial dataset, after removing population outliers, after removing children with only one measurement, and longitudinal outliers according to different cutoffs – for **height** **without decreasing height in dataset**. Brazilian Food and Nutrition Surveillance System (SISVAN), 2008–2017.

|  | **Initial dataset** | % | **Dataset after**  **removing**  **children with**  **only one**  **measurement** | % | **Dataset after removing LOs according to different cutoffs** | | | | | | | |
| --- | --- | --- | --- | --- | --- | --- | --- | --- | --- | --- | --- | --- |
|  |  |  |  |  | <-3 / >+3 | % | <-4 / >+4 | % | <-5 / >+5 | % | <-6 / >+6 | % |
| Boys |  |  |  |  |  |  |  |  |  |  |  |  |
| Severe  Stunting | 729,558 | 3.39 | 728,888 | 3.41 | 666,285 | 3.16 | 712,974 | 3.35 | 725,729 | 3.40 | 728,390 | 3.41 |
| Moderate  Stunting | 1,574,382 | 7.33 | 1,571,366 | 7.36 | 1,556,750 | 7.39 | 1,569,964 | 7.38 | 1,571,271 | 7.36 | 1,571,362 | 7.36 |
| Adequate | 19,185,615 | 89.28 | 19,054,165 | 89.23 | 18,839,008 | 89.45 | 18,987,851 | 89.27 | 19,037,728 | 89.23 | 19,051,376 | 89.23 |
| BIV | - | - | - |  | - | - | - | - | - | - | - | - |
| Missing | - | - | - |  | - | - | - | - | - | - | - | - |
| Total | 21,489,555 | 100.00 | 21,354,419 | 100.00 | 21,062,043 | 100.00 | 21,270,789 | 100.00 | 21,334,728 | 100.00 | 21,351,128 | 100.00 |
| Girls |  |  |  |  |  |  |  |  |  |  |  |  |
| Severe  Stunting | 582,939 | 2.62 | 582,351 | 2.63 | 516,652 | 2.37 | 565,369 | 2.57 | 579,014 | 2.62 | 581,847 | 2.63 |
| Moderate  Stunting | 1,391,059 | 6.25 | 1,388,241 | 6.28 | 1,373,033 | 6.30 | 1,386,830 | 6.30 | 1,388,143 | 6.28 | 1,388,237 | 6.28 |
| Adequate | 20,280,931 | 91.13 | 20,142,448 | 91.09 | 19,913,974 | 91.33 | 20,072,062 | 91.14 | 20,125,891 | 91.10 | 20,139,833 | 91.09 |
| BIV | - | - | - | - | - | - | - | - | - | - | - | - |
| Missing | - | - | - | - | - | - | - | - | - | - | - | - |
| Total | 22,254,929 | 100.00 | 22,113,040 | 100.00 | 21,803,659 | 100.00 | 22,024,261 | 100.00 | 22,093,048 | 100.00 | 22,109,917 | 100.00 |

**Table S5:** Prevalence of child growth indicator (WAZ) in the initial dataset, after removing population outliers, after removing children with only one measurement, and longitudinal outliers according to different cutoffs – for **weight**. Brazilian Food and Nutrition Surveillance System (SISVAN), 2008–2017.

|  | **Initial**  **dataset** | **%** | **Dataset after**  **removing**  **children with**  **only one**  **measurement** | **%** | **Dataset after**  **removing**  **children with**  **only one**  **measurement** | **%** | **Dataset after removing LOs according to different cutoffs** | | | | | | | |
| --- | --- | --- | --- | --- | --- | --- | --- | --- | --- | --- | --- | --- | --- | --- |
|  |  |  |  |  |  |  | <-3 / >+3 | % | <-4 / >+4 | % | <-5 / >+5 | % | <-6 / >+6 | % |
| Boys |  |  |  |  |  |  |  |  |  |  |  |  |  |  |
| Severe  Underweight | 289,385 | 1.17 | 231,194 | 0.99 | 227,140 | 0.98 | 188,207 | 0.82 | 210,593 | 0.91 | 220,821 | 0.95 | 224,851 | 0.97 |
| Moderate  Underweight | 787,856 | 3.19 | 746,050 | 3.19 | 738,207 | 3.19 | 709,458 | 3.11 | 730,854 | 3.17 | 736,395 | 3.18 | 737,749 | 3.19 |
| Adequate | 21,466,035 | 86.91 | 20,953,424 | 89.66 | 20,794,126 | 89.74 | 20,719,269 | 90.73 | 20,781,006 | 90.16 | 20,791,718 | 89.90 | 20,793,587 | 89.80 |
| Overweight | 1,714,033 | 6.94 | 1,438,213 | 6.15 | 1,412,005 | 6.09 | 1,219,635 | 5.34 | 1,326,967 | 5.76 | 1,377,522 | 5.96 | 1,399,275 | 6.04 |
| BIV | 401,966 | 1.63 | - | - | - | - | - | - | - | - | - | - | - | - |
| Missing | 39,356 | 0.16 | - | - | - | - | - | - | - | - | - | - | - | - |
| Total | 24,698,631 | 100.00 | 23,368,881 | 100.00 | 23,171,478 | 100.00 | 22,836,569 | 100.00 | 23,049,420 | 100.00 | 23,126,456 | 100.00 | 23,155,462 | 100.00 |
| Girls |  |  |  |  |  |  |  |  |  |  |  |  |  |  |
| Severe  Underweight | 245,969 | 0.97 | 200,784 | 0.83 | 197,667 | 0.82 | 165,335 | 0.70 | 184,932 | 0.77 | 193,168 | 0.81 | 196,164 | 0.82 |
| Moderate  Underweight | 737,655 | 2.90 | 703,726 | 2.91 | 696,740 | 2.90 | 671,768 | 2.84 | 690,598 | 2.89 | 695,220 | 2.90 | 696,369 | 2.90 |
| Adequate | 22,416,578 | 88.06 | 21,932,783 | 90.64 | 21,778,179 | 90.70 | 21,696,191 | 91.70 | 21,763,582 | 91.15 | 21,775,392 | 90.89 | 21,777,587 | 90.78 |
| Overweight | 1,614,511 | 6.34 | 1,359,451 | 5.62 | 1,337,378 | 5.57 | 1,127,351 | 4.76 | 1,238,680 | 5.19 | 1,294,214 | 5.40 | 1,320,003 | 5.50 |
| BIV | 387,168 | 1.52 | - | - | - | - | - | - | - | - | - | - | - | - |
| Missing | 54,226 | 0.21 | - | - | - | - | - | - | - | - | - | - | - | - |
| Total | 25,456,107 | 100.00 | 24,196,744 | 100.00 | 24,009,964 | 100.00 | 23,660,645 | 100.00 | 23,877,792 | 100.00 | 23,957,994 | 100.00 | 23,990,123 | 100.00 |
